# Chrysin Ameliorates Influenza Virus Infection in the Upper Airways by Repressing Virus-Induced Cell Cycle Arrest and Mitochondria-Dependent Apoptosis

**DOI:** 10.3389/fimmu.2022.872958

**Published:** 2022-03-31

**Authors:** Ying Liu, Xun Song, Chenyang Li, Hao Hu, Wanlin Li, Lu Wang, Jing Hu, Chenghui Liao, Hanbai Liang, Zhendan He, Liang Ye

**Affiliations:** ^1^Department of Pharmacy, Department of Immunology, International Cancer Center, Shenzhen University Health Science Center, Shenzhen, China; ^2^Department of Respiratory Medicine, Shenzhen University General Hospital, Shenzhen University, Shenzhen, China; ^3^College of Pharmacy, Shenzhen Technology University, Shenzhen, China

**Keywords:** chrysin, influenza virus, cell cycle, apoptosis, upper respiratory tract

## Abstract

Chrysin has been proven to possess antiviral properties, but the precise underlying anti-influenza mechanism and its anti-influenza efficacy *in vivo* are largely unclear. In this study, we investigated the involvement of chrysin in the blockade of cell cycle and apoptosis in distinct cell lines subjected to two H1N1 influenza A virus (IAV) strains, as well as its anti-IAV activity *in vivo*. Here, we found an early unidentified finding that chrysin strongly impeded IAV replication through a mechanism that was autonomous of innate antiviral immune activation and viral protein interaction. Surprisingly, chrysin can suppress IAV-induced cell cycle arrest in the G0/G1 phase by downregulating the expression levels of P53 and P21 while promoting Cyclin D1/CDK4 and Cyclin E1/CDK2 activation. Furthermore, chrysin dramatically inhibited the IAV-triggered mitochondrial apoptotic pathway by altering the balance of Bax/Bcl-xl and reducing caspase-9 and caspase-3 activation. Accumulated reactive oxygen species (ROS) reduction may contribute to the inhibitory role of chrysin in cell cycle arrest and apoptosis following IAV infection. Notably, chrysin preferably inhibited IAV replication in the upper respiratory tract, indicating that it might be a promising drug for restraining the spread of respiratory viruses.

## Introduction

Influenza viruses, which are members of the Orthomyxoviridae family, cause severe respiratory disease in humans, resulting in 300,000-500,000 fatalities each year worldwide (1, 2). Symptoms of the influenza virus are initially confined to the upper respiratory tract and are characterized by fever, runny nose, cough, headache, muscular pain, and exhaustion. They can further spread to the lower respiratory tract, causing severe or fatal viral pneumonia (1). When the influenza virus evades the host immune defense, it can be transmitted from infected individuals to healthy contacts. Influenza A virus (IAV) is an enveloped negative-sense single-strand RNA virus with eight RNA segments that encode viral nucleoprotein (NP), viral glycoproteins (hemagglutinin, HA), which facilitates viral entry, and neuraminidase (NA), which facilitates viral release, three RNA-dependent RNA polymerase subunits (polymerase acidic, PA) protein, the two polymerase basic proteins (PB1 and PB2), matrix protein (M1), surface protein (M2), the nonstructural protein NS1, and nuclear export protein (NEP; also known as NS2) (1, 2). The IAV HA and NA proteins are the most antigenically variable, with 18 HA subtypes and 11 NA subtypes (3). Although all IAV proteins, including HA, NA, NP, and RNA polymerases, are potential targets for developing anti-influenza therapeutic drugs, the most effective anti-influenza therapeutic candidates to date have been viral M2 ion channel (e.g., amantadine and rimantadine) and NA (e.g., Oseltamivir, zanamivir, and peramivir) inhibitors. However, current antiviral drugs are exposed to the rapid emergence of viral resistance due to the high frequency of IAV genome mutations (4–6), necessitating the development of new anti-influenza compounds.

Chrysin (5,7-dihydroxyflavone) is a natural flavone present in various medicinal plants, honey, and propolis that has been proven to have a wide range of pharmacological and biological effects (7, 8). Previous studies have reported that chrysin possesses antioxidant, anti-inflammatory, anti-cancer, anti-asthmatic, antibacterial, antiarthritic, antidiabetic, and neuroprotective functions, which have been validated in animal models of related diseases (7–10). Aside from these benefits, it was recently identified that chrysin may have antiviral properties. Zhang et al. revealed that chrysin and its 7-diisopropyl phosphate analogue can be effective against enterovirus 71 (EV71) replication without causing cytotoxicity by inhibiting viral 3C protease activity (11). In support of this result, chrysin exhibited potent antiviral action against coxsackievirus B3 (CVB3) *in vitro* and *in vivo*, protecting mice from CVB3-triggered viral pancreatitis (12). Furthermore, chrysin also has activity against herpes simplex virus types 1 (HSV-1) and 2 (HSV-2) *in vitro* (13). Comprehensive screening and comparison of the flavonoid compound’s library or flavonoid-enriched extracts highlighted the potential effect of chrysin in limiting IAV replication *in vitro* (14–17). However, in addition to a study showing that chrysin can disrupt the autophagy pathway to reduce IAV replication in cell culture (18), the precise mode of action and molecular mechanism of chrysin against IAV remain unclear, and no *in vivo* studies have been conducted to investigate the effect of chrysin against IAV.

In this study, we explored the anti-IAV effects of chrysin both *in vivo* and *in vitro* and revealed its potential mechanism of action for inhibiting IAV infection. We found that chrysin can suppress the infection of two H1N1 virus strains in both Madin-Darby Canine Kidney cells (MDCK cells) and adenocarcinomic human alveolar basal epithelial cells (A549 cells) by decreasing viral HA and NP synthesis but does not activate antiviral innate immunity. Our results further demonstrated that chrysin exerts its anti-IAV activity by an indirect interaction with IAV, resulting in a powerful therapeutic impact rather than a prophylactic effect. Furthermore, our study reveals previously unrecognized evidence that chrysin may effectively restrain G0/G1 phase cell cycle arrest by inhibiting ROS-mediated P53/P21 signaling while activating the Cyclin D1/CDK4 and Cyclin E1/CDK2 complexes. Meanwhile, chrysin restrained ROS-mediated apoptosis in a caspase-dependent pathway. Finally, chrysin treatment restrictively protects mice against IAV infection in the upper respiratory tract but not in the lower respiratory tract, indicating that chrysin might be a promising medication for limiting influenza virus transmission.

## Results

### Cytotoxic Effects of Chrysin on MDCK and A549 Cells

**Figure 1:**
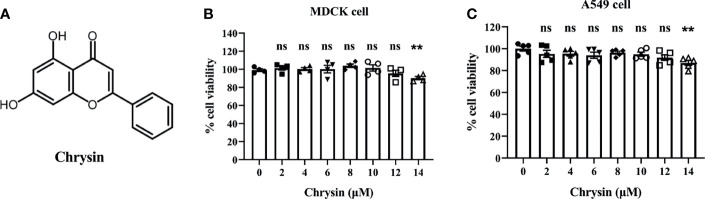
Chemical structure of chrysin and its cytotoxicity assay in MDCK and A549 cells. **(A)** Chemical structure of chrysin. MDCK **(B)** and A549 cells **(C)** were treated with increasing concentrations (0, 2, 4, 6, 8, 10, 12, 14 μM) of chrysin for 24 hours. Cell viability was assessed by the CCK-8 assay. Data was presented as mean ± SEM. **P < 0.01 compared with control (0 μM) by one-way ANOVA with Dunnett’s multiple comparisons test. ns, no significant difference. All data are representative of three independent experiments.

Chrysin, as illustrated by the chemical structure in **Figure 1A**, is a natural flavone abundant in many plant extracts, honey, and propolis that possesses a variety of biological actions. To investigate the toxicity of chrysin on cells, MDCK cells and A549 cells, two commonly utilized influenza virus susceptible cell lines, were treated with various concentrations of chrysin, followed by the cell counting kit 8 (CCK8) assay. The results demonstrated that chrysin had no cytotoxicity in both cell lines at 12 μM (**Figures 1B, C**). However, chrysin impacted cell viability up to 14 μM (**Figures 1B, C**), suggesting that subsequent studies at concentrations less than 12 μM chrysin are required.

### Chrysin Hampers Influenza A Virus Replication *In vitro*

**Figure 2:**
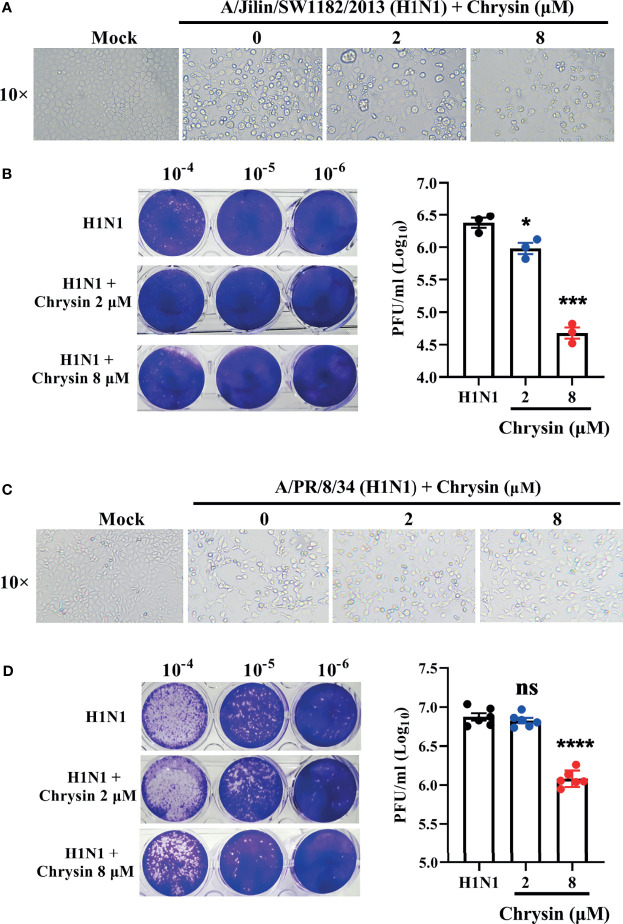
*In vitro* inhibitory activity of chrysin against H1N1 influenza virus infection. **(A, C)** Microscopy observations of virus-induced CPE reduction in MDCK cells by chrysin. A/Jilin/SW1182/2013 (H1N1) virus (MOI = 0.5) **(A)** or A/PR/8/34 (H1N1) (MOI = 0.5) **(C)** were incubated with chrysin (0, 2, or 8 μM) at 4°C for 30 min before infecting MDCK cells at 37°C for 2 hours. Subsequently, the viral inoculum was removed and replaced by adding chrysin (0, 2, or 8 μM). CPE was observed at 24 hours post-incubation by microscopy (original magnification × 10). **(B, D)** A/Jilin/SW1182/2013 (H1N1) virus (MOI = 0.5) **(B)** or A/PR/8/34 (H1N1) (MOI = 0.5) **(D)** was mixed with a different concentration of chrysin (0, 2, or 8 μM) at 4°C for 30 min, then infected with MDCK cells. Two hours after infection, the viral inoculum was removed, and the cells were treated with chrysin for 24 hours. The viral titer was detected by plaque assay and shown as a log value. Representative images of viral plaques in each histogram are shown (left panel). Data are expressed as the mean ± SEM of triplicate samples. *P < 0.05, ***P < 0.001, ****P < 0.0001 by one-way ANOVA with Dunnett’s multiple comparisons test when compared to the H1N1 control group. ns, no significant difference.

Influenza virus infection results in typical cell cytopathic effects (CPE) (1). To determine the antiviral activity of chrysin, a combination of chrysin and the influenza A virus (IAV) strain A/Jilin/SW1182/2013 (H1N1) was inoculated into MDCK cells and supplemented with chrysin for 24 hours. Here, we found that chrysin strongly diminished H1N1 virus-induced CPE formation on MDCK cells in a dose-dependent manner (**Figure 2A**). Moreover, plaque reduction assays the viral titers in the MDCK cell supernatant, indicating that chrysin can reduce H1N1 replication in the MDCK cell (**Figure 2B**). By using another IAV strain, A/PR/8/34 (H1N1), we found that 8 μM chrysin can similarly inhibit cytopathic effect (CPE) generation (**Figure 2C**) and viral titers (**Figure 2D**) in MDCK cells. These results reveal that chrysin has substantial antiviral efficacy against the H1N1 influenza virus.

### Chrysin Inhibits Influenza A virus HA and NP mRNA and Protein Synthesis But Does Not Elicit an Innate Antiviral Host Immunity

**Figure 3:**
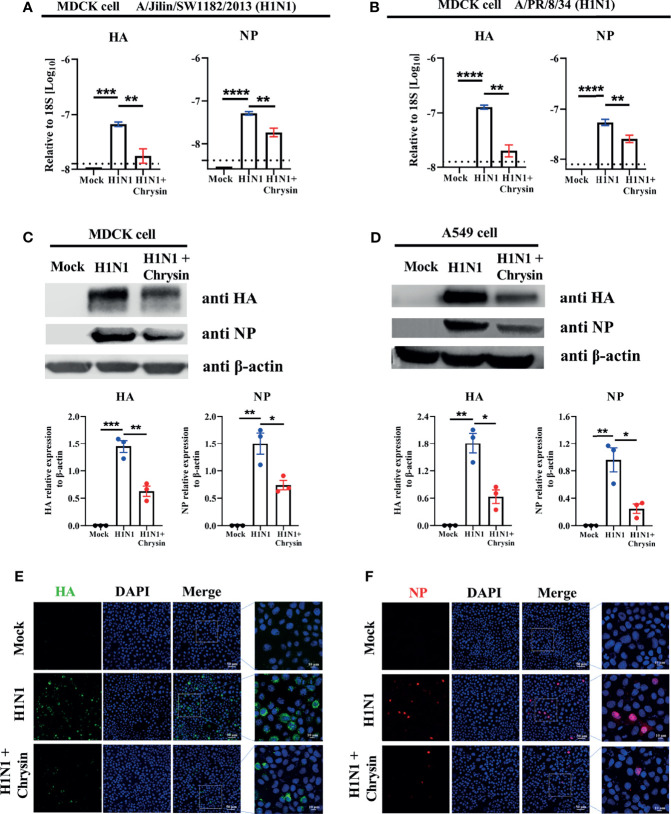
Chrysin suppresses H1N1 influenza virus replication *in vitro*. **(A, B)** MDCK cells were infected for 2 hours with A/Jilin/SW1182/2013 (MOI = 0.5) **(A)** or A/PR/8/34 (MOI = 0.5) **(B)** viruses with or without 8 μM chrysin, then the viral inoculum was removed and replaced by adding chrysin (8 μM) for 4 hours. HA and NP mRNA levels were determined by RT-qPCR and shown relative to the housekeeping gene 18s with a log value. **(C, D)** MDCK cells **(C)** or A549 cells **(D)** were infected with A/PR/8/34 (H1N1) virus at an MOI of 0.5 in the presence or absence of 8 μM chrysin for 2 hours, following which the cells were treated with chrysin for 4 hours. HA and NP protein levels were detected by western blotting and shown relative to β-actin with a regular value. Dara are expressed as the mean ± SEM. **(E, F)** MDCK cells were infected with the A/PR/8/34 (H1N1) virus at MOI of 0.5 for 2 hours, then cells were treated with 8 μM chrysin for 4 hours. Representative images of immunofluorescence staining in MDCK cells with antibodies against HA [**(E)**, green]) and NP [**(F)**, red]. The image in the white boxes on the left panels was further enlarged and shown on the right panels. Blue: positive staining of DAPI. All data are representative of three independent experiments, *P < 0.05, **P < 0.01 by unpaired two-tailed Student’s t-test. ***P < 0.001, ****P < 0.0001.

Given that chrysin can dampen IAV infection *in vitro* (18), we sought to see if the viral mRNA and protein levels of HA and NP were affected in cells supplemented with chrysin. To verify this hypothesis, MDCK cells were infected at an MOI of 0.5 with two IAV strains, A/PR/8/34 and A/Jilin/SW1182/2013, in the presence or absence of 8 μM chrysin for six hours before RT-qPCR analysis. Our results showed that the levels of viral HA and NP mRNA in MDCK cells were dramatically increased upon IAV infection, whereas the upregulated expression of viral HA and NP mRNA was abrogated in the presence of chrysin (**Figures 3A, B**). To test whether the effect of chrysin on viral mRNA expression also affect viral protein expression, the accumulation of viral HA and NP proteins was then examined in MDCK cells and A549 cells in the presence of chrysin following A/PR/8/34 infection by western-blot analysis of cell lysates, using antibodies for the HA and NP. When the IAV infection was supplemented with 8 μM chrysin in MDCK cells, HA and NP proteins decreased about 2-fold compared with the IAV control group (**Figure 3C**). Likewise, the production of HA and NP proteins was also significantly reduced in chrysin-treated A549 cells after IAV infection compared with IAV-infected control cells (**Figure 3D**). Consistent with the viral RNA and protein results, immunofluorescence analysis on IAV strain A/PR/8/34-infected MDCK cells to assess HA and NP levels found that HA and NP production were increased in IAV-infected cells but decreased in chrysin-treated infected cells (**Figures 3E, F**).

Since type I and type III interferon (IFN) systems are essential in the innate immune response against viruses (19), we intend to examine whether the inhibitory action of chrysin in response to IAV infection involves inducing type I and type III interferons as well as IFN-stimulated genes (ISG) expression. RT-qPCR was used to evaluate the innate antiviral gene expression of IFN (IFNα, IFN-β1, and IFN-λ) (**Supplementary Figure 1A**) and ISGs (Mx1, Isg15, and Oas2) (**Supplementary Figure 1B**) in IAV strain A/PR/8/34 infected A549 cells in the presence of chrysin, and it was determined that chrysin had no benefit in boosting mRNA expression of *IFNα*, *IFN-β1*, *IFN-λ*, *Mx1*, *Isg15*, *and Oas2* after IAV infection (**Supplementary Figure 1**). Taken together, these results demonstrate that chrysin suppresses IAV HA and NP production in an antiviral innate immunity-independent manner.

### Chrysin Exerts a Therapeutic Effect on Influenza A Virus Infection

**Figure 4:**
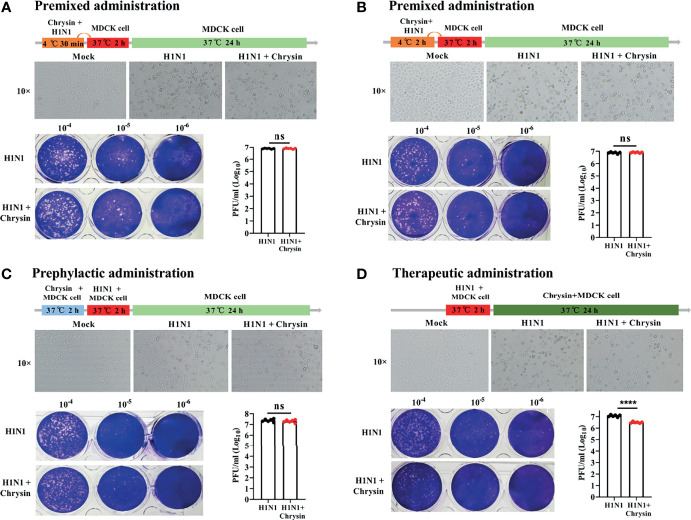
Premixed, prophylactic, and therapeutic efficacy of chrysin against the H1N1 influenza virus. **(A, B)** Premixed administration. Chrysin (8 μM) and A/PR/8/34 (H1N1) virus (MOI = 0.5) were mixed and incubated at 4°C for 30 min **(A)** or 2 hours **(B)**, and then the mixture was inoculated onto MDCK cells at 37°C. After 2 hours of incubation, the supernatant was removed and replaced by medium for culturing 24 hours. CPE was observed under a microscope, 10 × original magnification (upper panels). The Viral titer was detected by plaque assay (lower panels). **(C)** Prophylactic administration. MDCK cells were incubated with chrysin (8 μM) for 2 hours before being challenged with A/PR/8/34 (H1N1) virus (MOI = 0.5), after which the supernatant was removed and supplemented with fresh medium for culturing another 24 hours. CPE and viral titer were assessed using a microscope (upper panel) or a plaque test (lower panel), respectively. **(D)** Therapeutic administration. MDCK cells were infected with A/PR/8/34 (H1N1) (MOI = 0.5) at 37°C for 2 hours, then the supernatant was removed and cultured with new medium in the presence or absence of 8 μM chrysin for 24 hours before the CPE and viral titer were measured. All data are shown as the mean ± SEM of triplicate samples with a log value. When compared to the H1N1 control group, ****P < 0.0001 by unpaired two-tailed Student’s t-test. ns, no significant difference.

To investigate the antiviral effect mechanism of chrysin, we executed multiple models to evaluate the effectiveness of chrysin on influenza lifecycles, including premixed administration, prophylactic administration, and therapeutic administration. When chrysin was premixed with A/PR/8/34 (H1N1) for 30 minutes (**Figure 4A**) or 2 hours (**Figure 4B**), it failed to hinder H1N1 virus-induced CPE development (**Figures 4A, B**, upper panel) and limit virus replication (**Figures 4A, B**, lower panel), indicating that chrysin exerts its antiviral activity by indirect interaction with IAV. To confirm this hypothesis, we measured the binding capacity of chrysin and IAV related proteins by biolayer interferometry (BLI). As a result, BLI analyses revealed a poor affinity between chrysin and the HA, NA, M1, NS1, and NP, respectively (**Supplementary Figure 2**). In accordance with the findings of premixed administration, chrysin had no effect on depressing A/PR/8/34 virus-induced CPE generation (**Figure 4C**, upper panel) and viral titers (**Figure 4C**, lower panel) during prophylactic administration, implying that chrysin is ineffective throughout the early stages of the IAV lifecycle. It is worth noting that the inhibitory impact of chrysin on viral infection could be identified in therapeutic administration since the chrysin persists during the whole cycle of A/PR/8/34 virus replication (**Figure 4D**). Overall, these results suggest that chrysin confers an antiviral effect through indirectly interacting with IAV particles, and that chrysin has therapeutic potential when confronted with IAV infection.

### Chrysin Retards Influenza Virus-Induced Cell Cycle Arrest in the G0/G1 Phase

**Figure 5:**
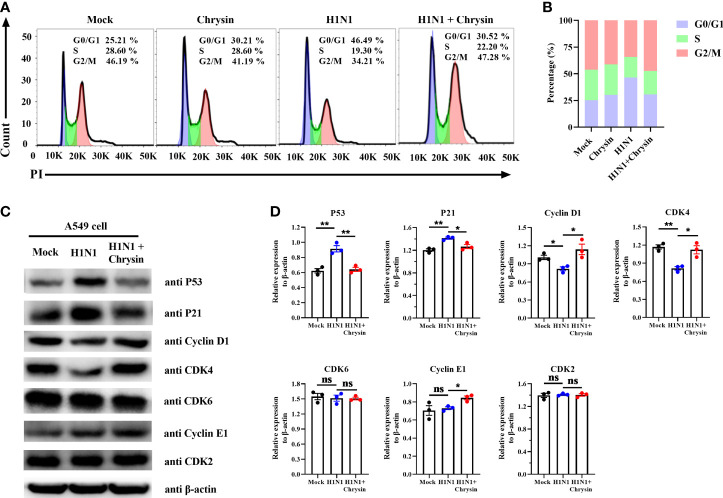
Chrysin retards H1N1 influenza virus-induced G0/G1 cell cycle arrest in epithelial cells. **(A)** A549 cells were infected with A/PR/8/34 (H1N1) virus (MOI = 0.5) in the presence or absence of chrysin for 2 hours, then the supernatant was removed and supplemented by fresh medium containing 8 μM chrysin for 10 hours stimulation. Cycle distribution was assessed using flow cytometry. **(B)** statistical charts of the cell population in G0/G1, S, and G2/M phases. **(C)** A549 cells were infected with A/PR/8/34 (H1N1) virus (MOI = 0.5) for 2 hours in the presence or absence of 8 μM chrysin, then the supernatant was discarded, and fresh medium containing 8 μM chrysin was added for 10 hours of treatment. Following that, the extracted protein was subjected to western blotting to determine the amounts of P53, P21, Cyclin D1, CDK4, CDK6, Cyclin E1, and CDK2. **(D)** Statistical analysis of the protein expression levels of P53, P21, Cyclin D1, CDK4, CDK6, Cyclin E1, and CDK2 in A549 cells. Protein loading was normalized based on β-actin and shown as a regular value. Data are expressed as the mean ± SEM. All data are representative of three independent experiments. *P < 0.05, **P < 0.01 by unpaired two-tailed Student’s t-test. ns, no significant difference.

Since influenza viruses can regulate the cell cycle, particularly at the G1/S transition, we wondered if the viral inhibition elicited by chrysin involves modulating cell cycle events. To address this question, A549 cells were infected with the influenza virus in the presence or absence of chrysin for flow cytometry investigation of cell cycle distribution. As expected, we were able to see an apparent accumulation of cells in the G0/G1 phase in A/PR/8/34 virus-infected cells, accompanied by reduced cell numbers in the S and G2/M phases (**Figure 5A**). The quantitative analysis showed that A/PR/8/34 virus-infected cells had a 21% increase in cell population in the G0/G1 phase as compared to mock-infected cells (**Figure 5B**). Surprisingly, chrysin can dampen the accumulation of cells in the G0/G1 phase induced by A/PR/8/34 virus infection, with a 15% reduction (**Figure 5B**). However, chrysin has a minor effect on S and G2/M phases (**Figures 5A, B**). These data indicate that chrysin can inhibit cell cycle arrest in the G0/G1 phase with IAV infection.

To further dissect the mechanism by which chrysin prevents G0/G1 cell cycle arrest, we investigated the DNA damage-mediated p53/p21 signaling pathway and checkpoint Cyclin/CDK complexes that govern the cell cycle. Western blot results revealed that IAV infection highly activated P53/P21 signaling pathways, resulting in elevated P53 and P21 protein levels, whilst chrysin clearly downregulated A/PR/8/34 virus-induced P53 and P21 protein expression (**Figures 5C, D**). It should be emphasized that A/PR/8/34 virus infection appears to selectively restrict cellular CDK4 and Cyclin D1 protein expression while having no influence on the expression of other checkpoint factors, including CDK6, CDK2, and Cyclin E1 (**Figures 5C, D**). Notably, treatment of chrysin in A/PR/8/34 virus-infected cells leads to increased expression of the proteins CDK4, Cyclin D1, and Cyclin E1 (**Figures 5C, D**). As a result of these findings, chrysin appears to effectively block G0/G1 phase cell cycle arrest by depressing P53/P21 signaling while promoting Cyclin D1/CDK4 and Cyclin E1/CDK2 activity.

### Chrysin Inhibits the Mitochondrial Apoptosis Pathway Following Influenza A Virus Infection

**Figure 6:**
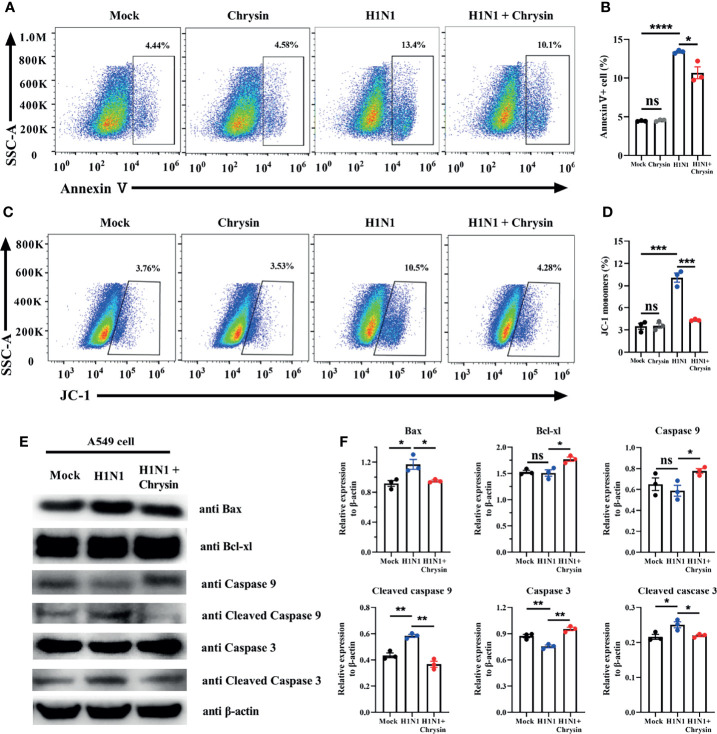
Chrysin inhibits the mitochondrial apoptosis pathway in H1N1 influenza virus-induced epithelial cells. A549 cells were infected with MOI of 0.5 A/PR/8/34 (H1N1) virus and chrysin for 2 hours, then the supernatant was removed and supplemented with fresh medium containing 8 μM chrysin for 10 hours of stimulation. **(A)** Cells were stained with Annexin V, and then analyzed by flow cytometry. **(B)** Statistical analysis of early Annexin V^+^ apoptosis ratio. **(C)** Representative histograms of JC-1 staining as measured by flow cytometry. **(D)** Statistical analysis of the monomeric JC-1 cell population. **(D)** Western blot of Bax, Bcl-xl, caspase 9, cleaved caspase 9, caspase 3, cleaved caspase 3. **(E)** Statistical analysis of the relative protein expression levels of Bax, Bcl-xl, caspase 9, cleaved caspase 9, caspase 3, cleaved caspase 3, protein loading were normalized based on β-actin and shown as a regular value. Data are representative of three similar experiments and are presented as mean ± SEM. *P < 0.05, **P < 0.01, ***P < 0.001, ****P < 0.0001 by unpaired two-tailed Student’s t-test. ns, no significant difference.

Influenza viruses can induce cell apoptosis (20). To determine whether IAV infection caused cell apoptosis can be reversed by chrysin, we performed Annexin V staining, a classic approach for detecting apoptosis. FACS analysis showed that A/PR/8/34 virus infection massively boosts the apoptotic cell population compared with the uninfected control in A549 cells (**Figures 6A, B**). Importantly, these early and late apoptotic events are obviously disrupted in A/PR/8/34 virus-infected A549 cells when chrysin is supplemented (**Figures 6A, B**). Previous studies have demonstrated that mitochondrial pathway-mediated intrinsic apoptosis is one of the most frequent types of apoptosis, involving the release of mitochondria-related signal factors. Cells with poor mitochondrial membrane potential (MMP) have been identified as apoptotic in general. Following A/PR/8/34 virus infection, A549 cells were treated with or without chrysin, and the changes in the MMP were examined utilizing the fluorescent mitochondrial membrane potential probe JC-1. FACS analysis showed a considerable loss in MMP after A/PR/8/34 virus infection, as evidenced by accumulations of cells stained with green fluorescence after exposure to furanodienone (**Figures 6C, D**). By contrast, chrysin can effectively abolish A/PR/8/34 virus-induced MMP reduction (**Figures 6C, D**).

To support the notion that the mitochondrial apoptotic pathway is responsible for chrysin suppressing A/PR/8/34 virus-mediated apoptosis, we investigated the expression of apoptosis-related proteins, particularly Bax and Bcl-xl. We found that chrysin dramatically reduced the level of pro-apoptotic Bax protein and markedly elevated the level of anti-apoptotic Bcl-xl protein in A549 cells after A/PR/8/34 virus infection (**Figures 6E, F**). In A/PR/8/34 virus-infected A549 cells, western blot analysis of caspase-9 and caspase-3 activation revealed that chrysin could restrain the levels of cleaved caspase-9 and cleaved caspase-3 (**Figures 6E, F**). These results suggest that chrysin-restrained apoptosis occurs in response to IAV infection *via* a mitochondrial-dependent apoptosis mechanism featuring Bcl-xl upregulation and caspase-dependent pathway activation as well as Bax downregulation.

### Chrysin Dampens ROS Production in Influenza A Virus Infection

**Figure 7:**
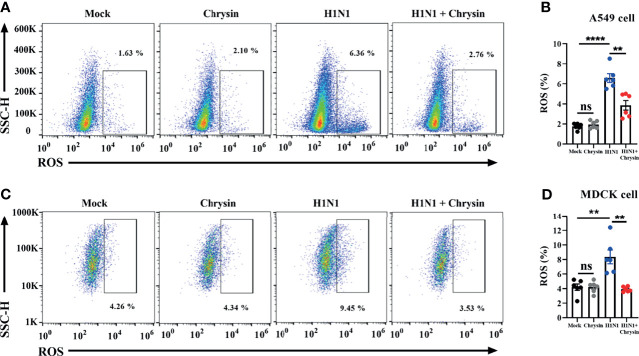
Chrysin abrogates H1N1 influenza virus-induced ROS production. A549 cells or MDCK cells were infected for 2 hours with a MOI of 0.5 A/PR/8/34 (H1N1) virus and 8 μM chrysin, then the supernatant was withdrawn and replaced with medium containing 8 μM chrysin for a total of 10 hours of treatment. A549 cells **(A, B)** and MDCK cells **(C, D)** were stained by DCFH-DA for flow cytometry analysis. Data are presented as mean ± SEM of three independent experiments with similar results. **P < 0.01, ****P < 0.0001 , by unpaired two-tailed Student’s t-test. ns, no significant difference.

The ROS produced by mitochondria plays a crucial role in the control of apoptosis and cell cycle arrest. We thus investigated if the anti-apoptotic and cell cycle regulatory activities of chrysin were a result of ROS modulation within IAV-infected cells. The DCFH-DA fluorescent probe was used to measure the change in intracellular ROS levels in chrysin-treated A549 cells and MDCK cells after A/PR/8/34 virus infection. As anticipated, A/PR/8/34 virus infection markedly increased ROS production in A549 cells compared with uninfected control (**Figure 7A**). Likewise, the ROS level was significantly elevated in A/PR/8/34 virus-infected MDCK cells when compared to that of the uninfected control (**Figure 7B**). Of note, chrysin treatment remarkably reversed the A/PR/8/34 virus-induced ROS level in A549 cells and MDCK cells (**Figures 7A, B**). These results reveal that intracellular ROS generation is involved in the inhibitory effects of chrysin on IAV-induced apoptosis and cell cycle arrest.

### Intragastrical Administration of Chrysin Inhibits Influenza Virus Infection in the Upper Respiratory Tract

**Figure 8:**
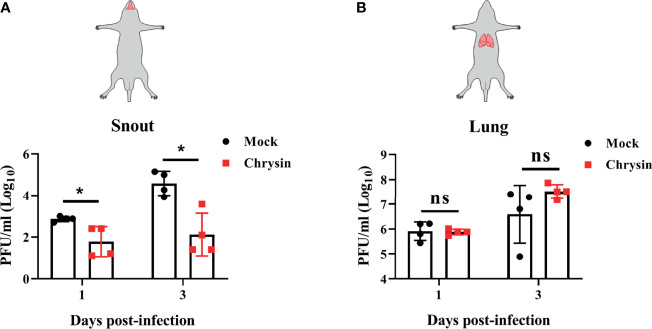
Intragastrical administration of chrysin blocks H1N1 influenza virus replication in the upper airways. C57BL/6 mice were intranasally infected with18 PFU A/PR/8/34 (H1N1) virus in a total volume of 40 μl for 2 hours, then animals were administered chrysin (100 mg/kg) daily by gavage before being sacrificed. Snout **(A)** and lung **(B)** samples were harvested on days 1 and 3 following infection for viral titer analysis. Data are expressed as the mean ± SEM of three independent experiments with a log value. *P<0.05 by unpaired two-tailed Student’s t-test. ns, no significant difference.

To assess the protective efficacy of chrysin treatment on influenza-infected mice, C57BL/6 mice were challenged intranasally with a lethal dose of A/PR/8/34 virus before receiving an intraperitoneal or intragastric dosage of 100 mg/kg chrysin daily, while body weight and survival rate were monitored. It is disappointing that intraperitoneal chrysin treatment did not reverse IAV-induced weight loss and did not extend the survival period or improve the survival rate of mice post IAV infection (**Supplementary Figures 3A, B**). Consistent with these results, intragastric administration of chrysin also failed to protect mice against IAV challenge (**Supplementary Figures 3C, D**). We next sought to investigate whether the antiviral role of chrysin was limited to the upper respiratory tract rather than the lower respiratory tract, failing to face the challenge of IAV. To verify this hypothesis, C57BL/6 mice were administered 100 mg/kg chrysin intragastrically every day after intranasal A/PR/8/34 virus infection, and viral titers in the snout and lungs were measured at days 1 and 3 post-infection. We found at least 10-fold less infectious IAV in the snouts of chrysin-treated mice compared with untreated control between days 1 and 3 post-infection (**Figure 8A**), but the viral tiers in the lungs were not affected by chrysin at the indicated time points (**Figure 8B**). These results indicate that chrysin treatment potently suppressed IAV replication in the upper airways, which might be beneficial for restraining viral transmission.

## Discussion

Previous studies revealed that chrysin has considerable antiviral activity when exposed to EV71, CVB3, and HSV infection (11–13). However, little is known about the underlying mechanism of action and its antiviral efficacy *in vivo* following influenza virus infection. In this work, we reported previously unrecognized aspects that chrysin could not directly act on influenza A virus (IAV) associated proteins but instead suppresses IAV-induced intracellular ROS generation, which further dampens cell cycle G0/G1 arrest and mitochondrial apoptotic pathways. Through these mechanisms, chrysin profoundly inhibits IAV infection both in cultured cells and in mice, irrespective of innate antiviral immunity. Interestingly, the therapeutic efficacy of chrysin can selectively block IAV replication in the upper airways but not in the lungs, showing the potential value of suppressing IAV transmission.

Since flavonoids are recommended as a dietary element in the prevention and treatment of influenza viruses (14, 17, 21), we extend the concept of whether and how chrysin, one of the natural flavonoids, contributes to IAV protection. Our results showed that chrysin, like other flavonoids, has substantial antiviral activity against IAV infection in MDCK cells and A549. The strong inhibitory role of chrysin in IAV infection is linked to a reduction in viral HA and NP transcription and protein levels. These findings are consistent with an earlier investigation, which reported that chrysin suppressed viral HA production as well as viral replication, and that its anti-IAV effectiveness was marginally better than that of the NA inhibitor, Oseltamivir (18). Based on a protein-ligand molecular computer prediction study which suggested chrysin as a possible inhibitor of influenza H1N1 virus neuraminidase (22), we evaluated whether the anti-IAV activity of chrysin was attributable to interaction with viral proteins. Inconsistent with expectations, our BLI analysis indicated that chrysin has a low affinity for NA as well as other viral proteins including HA, M1, NS1, and NP. The investigation of IAV lifecycles supported these conclusions, showing that chrysin had no effect in the early phases of the IAV lifecycle and only displayed a therapeutic effect, not a preventive or control role. These results suggest that the inhibitory impact of chrysin on IAV is an indirect mechanism that does not involve direct interaction with IAV surface proteins or particles, which differs from other flavonoids such as kaempferol, quercetin, and naringenin (22, 23). As a result, one or more intracellular signaling pathways were likely related to the anti-influenza activity of chrysin.

The interferons (type I interferon (namely, IFN-α/β) and type III interferon (namely, IFN-λ)) induced by viruses are the first innate immunity line of defense against virus infection (19). IFN-α/β and IFN-λ do not directly combat viruses but instead activate JAK-STAT signaling to induce the expression of hundreds of interferon-stimulated genes (ISGs), including Mx1, Isg15, and Oas2 (24). Therefore, increasing IFN-α/β or IFN-λ production is an effective strategy for controlling IAV infection. The screening of flavonoids-based anti-influenza virus drugs showed that flavonoid compounds had the capability to improve IFN production (25–27). Our data, however, show that chrysin has no effect on increasing IFN-α/β or IFN-λ expression in the inclusion or exclusion of IAV infection. Similarly, the expression of typical ISGs in MDCK cells remained constant after chrysin treatment regardless of whether there was an IAV infection. These data highlight that the anti-influenza features of chrysin are determined by an independent mechanism of host innate immune responses.

IAV replication can induce cell cycle arrest in the G0/G1 phase, promoting viral protein expression and progeny virus production (28). P53 has been recognized as a multifunctional transcription factor involved in cell cycle modulation (29). IAV-induced P53 activation can raise p21 expression, which acts as an inhibitor, binding to and limiting the checkpoint factor Cyclin-CDK complex activity, hence blocking the G1/S transition (28). Cyclin D1 facilitates Cyclin D-CDK4/6 activity, while Cyclin E1 benefits cyclinE1-CDK2 activity (30). Thus, interrupting P53/P21 signaling-mediated Cyclin-CDK complex activity to disrupt cell cycle transition from G0/G1 phase to S phase events is favorable for halting IAV infection. Consistent with this theory, our data showed that IAV triggers cell cycle arrest in the G0/G1 phase in epithelial cells, which can be effectively reversed by chrysin, along with reduced P53 and P21 production. Furthermore, chrysin markedly increases the levels of Cyclin D1 and E1 and CDK4 in IAV-infected epithelial cells, implying a particular regulation of activating cyclin D1-CDK4 and cyclin E1-CDK2 in chrysin treated cells upon IAV infection. Therefore, chrysin may dampen IAV-induced P53/P21 signaling pathway activation, which restrains cyclin D1-CDK4 and cyclin E1-CDK2 activity and thus inhibits IAV-induced cell cycle arrest that occurs in the G0/G1 phase.

**Figure 9:**
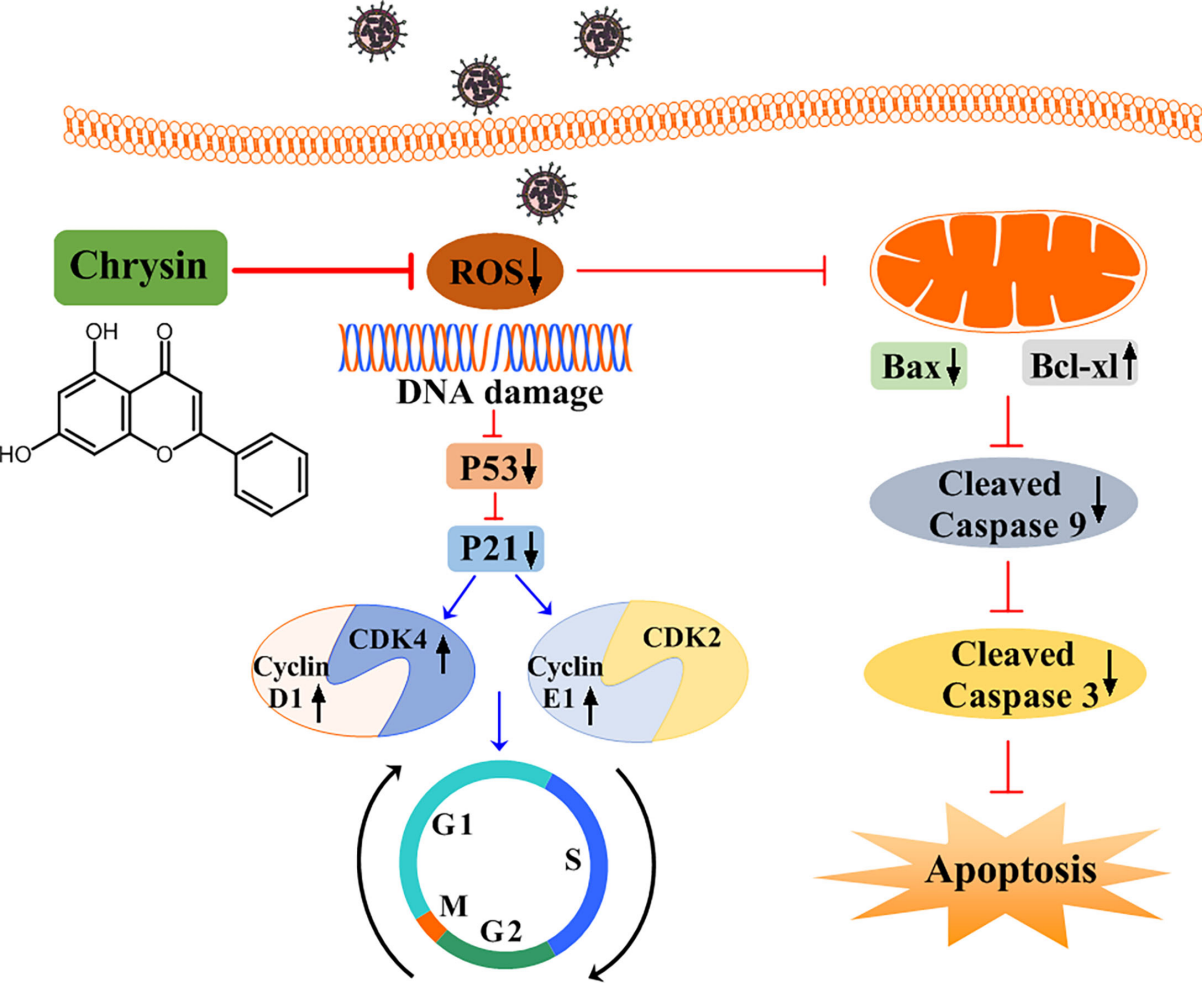
A schematic diagram of the role of chrysin in suppressing influenza virus infection by blocking G0/G1 cell cycle arrest and the mitochondrial apoptosis pathway. Chrysin restrains influenza virus infection by disrupting ROS-mediated dual signaling pathways, including the suppression of p53 and p21, which promotes the activation of CDK4, Cyclin D1, and Cyclin E1, culminating in the prevention of cell cycle arrest at the G0/G1 phase. Alternatively, chrysin can reduce ROS-mediated Bax while increasing Bcl-xl, which in turn blocks mitochondrial apoptosis by inhibiting cleaved-caspase 9 and cleaved-caspase 3.

In addition to promoting cell cycle arrest, IAV can trigger host cellular apoptosis in ways that facilitate efficient viral replication and propagation (20). Apoptotic occurs *via* two main pathways: the extrinsic apoptotic pathway and the intrinsic apoptotic pathway, both of which are dependent on caspase-9 activation (31). We noticed that chrysin inhibits IAV-induced cleaved caspase-9 levels in epithelial cells. Moreover, chrysin diminished the IAV-activated levels of caspase-3, a caspase-9 downstream signaling effective molecule in the intrinsic apoptotic cascade. These findings indicate that chrysin could dampen the IAV-induced intrinsic apoptotic pathway, thereby improving the survival of host cells. Prior studies have shown that the proapoptotic member Bax contributes to activation of the intrinsic apoptotic pathway, while the antiapoptotic member Bcl-xl can heterodimerize with proapoptotic proteins to prevent the apoptotic process (32, 33). When the Bax/Bcl-xl ratio balance was upset, the MMP was disrupted, triggering the activation of caspase cascades and, eventually, apoptosis. In the present study, we confirmed that decreased Bax but increased Bcl-xl protein with a concomitant reduction of MMP in chrysin-treated cells following IAV infection, suggesting chrysin alters the ratio of proapoptotic and antiapoptotic proteins to drive the mitochondria-mediated apoptotic pathway. ROS accumulation, which results in DNA damage, is essential for cell cycle progression and apoptosis. ROS can either enhance the permeability of the outer mitochondrial membrane, ultimately activating the intrinsic apoptotic pathway, or activate P53 signaling, subsequently regulating the cell cycle. Recent work reported that chrysin protects against aluminum phosphide-induced oxidative stress and mitochondrial damage by suppressing ROS and MMP production (34). Consistently, our results showed that chrysin treatment greatly reduced IAV-induced ROS levels, which might translate to the blockage of IAV-triggered intrinsic apoptotic pathways and the cell cycle arrest at G0/G1 phage (**Figure 9**).

Although flavonoids have been shown to attenuate IAV-induced acute lung injury (17, 27), the *in vivo* anti-influenza efficacy of chrysin remains unclear. Unlike other flavonoids that can defend mice from IAV infection, chrysin cannot withstand a lethal dose of IAV challenge in mice, regardless of whether it is delivered intraperitoneally or intragastrically. Unexpectedly, chrysin treatment can reduce IAV titers in the snout but not the lungs of mice. Because the influenza virus is readily transmitted in the community, restricting IAV transmission from infected people to uninfected contacts is an effective strategy for preventing respiratory virus infection (35, 36). The unique properties of chrysin limit viral replication in the upper respiratory tract, hinting that chrysin has the potential to control transmission of respiratory viruses such as IAV and severe acute respiratory syndrome coronavirus 2 (SARS-CoV-2) in the future. To support this hypothesis, a suitable animal respiratory transmission model needs to be established to assess the antiviral transmission role of chrysin.

In summary, our study identifies the anti-influenza effects of chrysin *in vitro* and *in vivo*. We found that chrysin dramatically blocks IAV-induced G0/G1 phase arrest by reducing ROS generation, which in turn downregulates P53 and P21 expression, hence restraining cyclin D1-CDK4 and cyclin E1-CDK2 activity (**Figure 9**). Alternatively, chrysin can limit IAV-triggered cell apoptosis through a ROS-dependent mitochondrial pathway in combination with increased Bcl-xl expression, decreased Bax expression, and reduced caspase-9 and caspase-3 activation (**Figure 9**). This work stresses the importance of chrysin in controlling IAV replication in the upper airways, which might be utilized to develop chrysin as a potential drug for inhibiting respiratory virus transmission.

## Materials and Methods

### Cells, Viruses, and Mice

Madin-Darby canine kidney (MDCK) cells and Adenocarcinomic human alveolar basal epithelial cells (A549 cells) from ATCC were cultured at 37 °C under 5% CO_2_ in Dulbecco’s Modified Eagle Medium (DMEM) (Gibco: C11995500BT) supplemented with 10% inactivated fetal bovine serum (FBS) (Gibco: 10270106) and 1% penicillin/streptomycin (Gibco: 15140-122).

Influenza A viruses, A/PR/8/34 and A/Jilin/SW1182/2013, were propagated in MDCK cells and titrated as previously described (35). Viruses were stored at -80 °C until they were used in subsequently experiments.

Female C57BL/6 mice (6-8 weeks) were purchased from GemPharmatech Co., Ltd. All mice were bred at the Shenzhen University animal facility under specific pathogen-free (SPF) conditions. The institutional Animal Care and Use Committee at Shenzhen University authorized all animal experiments.

### Cytotoxicity Assay

MDCK and A549 cells were seeded in 96 well culture plates at 1.0 × 10^4^ cell/well and incubated at 37°C overnight with 5% CO_2_. Chrysin (Meryer, F21744-5G) was applied to the cells and incubated for 24 hours at the indicated concentrations (2, 4, 6, 8, 10, 12, 14 μM). Then the CCK-8 reagent (Dojindo, CK04) was added at 10 μl per well for 2 hours. The solution absorbance was measured at 450 nm by a microplate reader (Rayto, China).

### Cytopathic Effect Assay

MDCK cell were plated in 12-well plates at a density of 2.0 ×10^5^ cells/well and incubated overnight at 37°C with 5% CO_2_. The next day, the various concentrations of chrysin and A/Jilin/SW1182/2013 (H1N1) (MOI = 0.5) or A/PR/8/34 (H1N1) (MOI = 0.5) were mixed at 4°C for 30 min before being added to MDCK cells at 37°C for 2 hours. The supernatant was then discarded, and the cells were washed twice with PBS (Solarbio, P1010). Finally, different quantities of chrysin were applied to the cells and they were cultured at 37°C for 24 hours. The cytopathic effect was observed under a microscope.

### Flow Cytometry

MDCK cells and A549 cells were treated with a combination of A/PR/8/34 (H1N1) (MOI = 0.5) and chrysin (8 μM) for 2 hours at 37°C. Then, the medium was removed, and the cells were treated with chrysin (8 μM) alone for a total of 10 hours at 37°C. Flow cytometric analysis was performed using the following kits: mitochondrial membrane potential assay kit with JC-1 (Beyotime, C2006); Reactive Oxygen Species (ROS) Assay Kit (Beyotime, S0033S); Annexin V-FITC Apoptosis Detection Kit (BD Bioscience, 556547); Cell Cycle and Apoptosis Analysis Kit (Beyotime, C1052). Cells were collected, which were then stained according to the manufacturer’s instructions. Flow cytometric analysis was performed using an Attune™ NxT Acoustic Focusing Cytometer (Thermo Scientific, America) and the data was analyzed with Flowjo software.

### Immunofluorescence Staining

MDCK cells (2.0 × 10^5^ cells) were treated with the mixture of A/PR/8/34 (H1N1) (MOI = 0.5) and Chrysin (8 μM) for 2 hours at 37°C. The medium was discarded and treated with fresh medium containing 8 μM chrysin at 37°C for 4 hours. Cells were fixed in a 4% paraformaldehyde solution (Beyotime, P0099) for 15 minutes and permeabilized in 0.2% Triton X-100 (Sigma, X100) for 15 minutes. After blocking with 3% bovine serum albumin (Sigma, A1933) for 1 hour, cells were incubated with anti-HA antibody (Thermofisher, PA5-34929) or anti-NP antibody (Thermofisher, MA5-35899) overnight at 4°C. Cells were incubated with an Alexa Fluor 488 tagged anti-rabbit IgG antibody (Cell signaling technology, ab150077) or an Alexa Fluor 647 tagged anti-mouse IgG antibody (Cell signaling technology, ab150115) at 37°C for 1 hour. After washing with PBS, the cells were stained with 1 µg/ml DAPI (Beyotime, C1006) for 20 minutes and analyzed using ZEISS ZEN digital microscope software.

### Plaque Reduction Assay

Confluent MDCK cells seeded into 12-well plates were washed with PBS and inoculated with a gradient dilution of virus solution [Opti-MEM (Thermo Fisher Scientific, 31985070) with 0.3% BSA and 2.5 μg/ml TPCK-treated trypsin (Sigma, T1426)] for 2 hours. Cell monolayers were overlaid with 1.5 ml of semisolid medium (1.5% CMCNa: DMEM = 1:1, with 0.3% BSA and 2.5 μg/ml TPCK-treated trypsin) and incubated at 37°C for 30 hours after the virus inoculums were removed, followed by fixation of the cell monolayers with 4% paraformaldehyde and staining with 0.2% (w/v) crystal violet (Sigma, C0775). The size of plaques was counted and measured using Image J software.

### Quantitative Reverse Transcription-Polymerase Chain Reaction

Cells were harvested for extraction of total RNA using Trizol reagent (Takara, 9109) according to the manufacturer’s protocol. cDNA synthesis was carried out using the RevertAid First Strand cDNA Synthesis Kit (Thermo Fisher, K1622). After reverse transcription, qPCR amplification was performed using Tip Green qPCR SuperMix (Transgen, China, AQ141-01) on a CFX96 Real-Time PCR System (Bio-Rad, USA). The sequences of the primers are as follows: *18s*: forward: 5’-CCCCTCGATGCTCTTAGCTG-3’, reverse: 5’-CTTTCGCTCTGGTCCGTCTT-3’; *Ha*: forward: 5’-TATTCGTCT

CAGGGAGCAAAAGCAGGGG-3’, reverse: 5’ ATATCGTCTCGTATTAGTAGAAACAAGGGTGTTTT-3’; *Np*: forward: 5’-TATTCGTCTCAGGGAGCAAAAGCAGGGTA-3’, reverse: 5’-ATATCGTCTCGTATTAGTAGAAAC

AAGGGTATTTTT-3’; *Ifnα*: forward: 5’-AGAAGGCTCCAGCCATCTCTGT-3’, reverse: 5’-TGCTGGTAGAGT

TCGGTGCAGA-3’; *Ifnβ1*: forward: 5’-CTTGGATTCCTACAAAGAAGCAGC-3’, reverse: 5’-TCCTCCTTCTG

GAACTGCTGCA-3’; *Ifnλ*: forward: 5’-AACTGGGAAGGGCTGCCACATT-3’, reverse: 5’-GGAAGACAGGA

GAGCTGCAACT-3’; *Mx1*: forward: 5’-GGCTGTTTACCAGACTCCGACA-3’, reverse: 5’-CACAAAGCCTGG

CAGCTCTCTA-3’; *Isg15*: forward: 5’-CTCTGAGCATCCTGGTGAGGAA-3’, reverse: 5’-AAGGTCAGCCAG

AACAGGTCGT-3’; *Oas2*: forward: 5’-GCTTCCGACAATCAACAGCCAAG-3’, reverse: 5’-CTTGACGATTTT

GTGCCGCTCG-3’. PCR programs consist of 95°C for 10 minutes, 40 cycles of 95°C for 15 seconds, and 30 seconds at 61°C. The mRNA expression of target genes in each group was determined by the 2^−ΔCt^ method relative to the expression of 18S.

### The Methods of Three Modes of Medication

MDCK cells were seeded into 12-well plates and cultured at 37°C under 5% CO_2_ for 24 hours.

*For premixed administration*, chrysin (8 μM) and A/PR/8/34 (H1N1) (MOI = 0.5) were mixed at 4°C for 30 min or 2 hours before being added to MDCK cells for 2 hours of treatment at 37°C. The supernatant was discarded and washed twice with PBS. Cells were cultured for 24 hours at 37°C. The supernatant was collected for a plaque assay to determine the viral titer.

*For prophylactic administration*, chrysin (8 μM) was added to stimulate MDCK cells for 2 hours, then the cells were infected by A/PR/8/34 (H1N1) (MOI = 0.5) for 2 hours. The medium was removed and replaced by fresh medium at 37°C for 24 hours. The supernatant was collected, and the virus titer was detected by plaque assay.

*For therapeutic administration*, MDCK cells were infected with A/PR/8/34 (H1N1) (MOI = 0.5) at 37°C for 2 hours. The medium was removed and replaced by fresh medium containing 8 μM chrysin at 37°C for 24 hours. The supernatant was collected, and the virus titer was determined by plaque assay.

### Biolayer Interferometry

The affinity of chrysin and His-tagged protein of influenza A virus (IAV) was determined using Ni-NTA biosensors (Forte Bio, 18-0029), onto which 1 μM of protein of influenza virus was in running buffer (PBS, 0.02% Tween-20, and 0.1% BSA) was loaded. IAV HA protein (Sino Biologicol, 40673-V08H), NA protein (Sino Biologicol, 40197-V07H), M1 protein (Sino Biologicol, 40010-V07E), NP protein (Sino Biologicol, 11675-V08B), and NS1 protein (Sino Biologicol, 40011-V07E). Chrysin was diluted to 500 μM, 250 μM, and 125 μM in the running buffer. First, the Ni-NTA sensor tips were hydrated in the buffer for 10 minutes prior to use. Then sensor baselines were equilibrated in the running buffer for 30 seconds (the initial baseline). Next, the protein was loaded until saturated for 240 seconds (the loading phase), and the sensor was washed for 120 seconds in the running buffer (baseline phase). The sensors were immersed in wells containing chrysin for 240 seconds (the association phase), followed by immersion in running buffer for another 240 seconds (the dissociation phase). Curve fitting was performed using a 1:1 binding model and the ForteBio data analysis software.

### Western Blot Analysis

A549 cells were washed with ice-cold PBS, and lysed with RIPA buffer (Solarbio, R0010) for protein extraction, and then quantified using the BCA assay kit (Beyotime, P0012) according to the manufacturer’s instructions. They then added 5 × protein loading buffer (Meilunbio, MA0003-D) to sample and were boiled at 95°C for 5 minutes. An equivalent quantity of protein samples was loaded onto a 10% sodium dodecyl sulfate-polyacrylamide gel electrophoresis gel (Beyotime, P0012AC) and subsequently transferred to a polyvinylidene fluoride (PVDF) membrane (Merck Millipore, IPVH15150). Then on-specific binding was blocked with 5% skimmed milk (BioFroxx, 1172GR500), and it was incubated with primary antibodies against P53 Polyclonal antibody, P21 Rabbit Polyclonal antibody (Proteintech, 10355-1-AP), Cyclin D1 Rabbit Polyclonal antibody (Proteintech, 60186-1-lg), CDK4 Polyclonal antibody (Proteintech, 11026-1-AP), CDK6 Polyclonal antibody (Proteintech, 14052-1-AP), Cyclin E1 Rabbit Polyclonal antibody (Proteintech, 11554-1-AP), CDK2 Polyclonal antibody (Proteintech, 10122-1-AP), Bax antibody (Cell signaling technology, 2772), Bcl-xl rabbit polyclonal antibody (Proteintech, 26967-1-AP), Caspase 3 antibody (Cell signaling technology, 9662), Caspase 9 polyclonal antibody (Proteintech, 10380-1-AP), H1N1 NP Monoclonal Antibody (Thermo Fisher, GT1236), H1N1 HA Polyclonal Antibody (Thermo Fisher, PA5-34929), β-actin antibody (Abcam, ab8226) overnight at 4°C. After washing with Tris-buffered solution containing 0.05% Tween 20, the blots were incubated with secondary antibodies (Abcam, ab6721/ab6728) for two hours at room temperature. After five additional washes, the protein bands were visualized using an ECL reagent kit (Solarbio, PE0010) based on the manufacturer’s instructions (Carestream, USA).

### Virus Infection in Mice

Female C57BL/6 mice (6-8 weeks old) were acclimated for 7 days before experiments. Mice were intranasally infected with 18 PFU A/PR/8/34 (H1N1) viruses in a total volume of 40 μl for 2 hours before receiving chrysin (100 mg/kg) daily by either gavage or intraperitoneal injection. Weight loss and survival were monitored on a daily basis until the animals were scarified after they had lost 25% of their initial body weight. Snout and lung samples were collected on days 1 and 3 following infection for plaque assays in several repeats.

### Statistical Analysis

Statistical analysis was performed using GraphPad Prism 8.0. The experimental results were conducted out in at least three replicates. All data are shown as means with standard deviations (SEM). The data were statistically analyzed using unpaired two-tailed Student’s t-test or one-way ANOVA with Dunnett’s multiple comparisons test. P values are considered to indicate a significant difference, as shown in the figures as follows: *p<0.05; **p<0.01; ***p<0.001; ****P<0.0001.

## Data Availability Statement

The original contributions presented in the study are included in the article/**Supplementary Material**. Further inquiries can be directed to the corresponding authors.

## Ethics Statement

The animal study was reviewed and approved by The Institutional Animal Care and Use Committee at Shenzhen University.

## Author contributions

LY and YL designed experiments, YL, XS, YL, HH, WL, LW, JH, ChL, and HL performed experiments. YL, XS, and LY performed data analysis and prepared figures. LY wrote the manuscript. ZH, and LY edited the manuscript and supervised the project. All authors contributed to the article and approved the submitted version.

## Funding

This work was supported by grants from the National Nature Science Foundation of China (NSFC, 32170937, 31670360, and U1702286), Guangdong Basic and Applied Basic Research Foundation (2020A1515110410, 2021A1515010917, 2019B1515120029, and 2020A1515111169), Natural Science Foundation of the Education Department of Guangdong Province (2020KZDZX1172), Shenzhen Science and Technology Program (RCBS20200714114958310, 20200803131335002, and 20200813201847001), Guangdong Medical Science and Technology Research Foundation (A2021336, 817/000655), Shenzhen Peacock Plan Project (RC00325, 827/000569, 827/000655), and SZU Top Ranking Project (86000000210, 860000002110131).

## Conflict of Interest

The authors declare that the research was conducted in the absence of any commercial or financial relationships that could be construed as a potential conflict of interest.

## Publisher’s Note

All claims expressed in this article are solely those of the authors and do not necessarily represent those of their affiliated organizations, or those of the publisher, the editors and the reviewers. Any product that may be evaluated in this article, or claim that may be made by its manufacturer, is not guaranteed or endorsed by the publisher.

## Abbreviations

IAV, influenza A virus; P53, tumor protein P53; p21, cyclin-dependent kinase inhibitor 1; CDK, cyclin-dependent kinase; Bax, Bcl-2-associated X protein; Bcl-xl, B-cell lymphoma-extra large; ROS, reactive oxygen species; NP, nucleoprotein; HA, hemagglutinin; NA, neuraminidase; PA, polymerase acidic; PB, polymerase basic protein; M1, matrix protein; M2, surface protein; NS1, nonstructural protein; NEP, nuclear export protein; EV71, enterovirus 71; CVB3, coxsackievirus B3; HSV-1, herpes simplex virus type 1; HSV-2, herpes simplex virus type 2; MDCK cell, madin-darby canine kidney cell; A549 cell, adenocarcinomic human alveolar basal epithelial cell; CCK8, cell counting kit 8; CPE, cytopathic effect; IFN, interferon; ISG, IFN-stimulated gene; MX1, mx dynamin like GTPase 1; ISG15, interferon-stimulated gene 15; OAS2, 2’-5’-Oligoadenylate Synthetase 2; JC-1, mitochondrial membrane potential probe; MMP, mitochondrial membrane potential; DCFH-DA, dichloro-dihydro-fluorescein diacetate; JAK, janus kinase; STAT, signal transducer and activator of transcription.
