# Supplementary material for: Chrysin Ameliorates Influenza Virus Infection in the Upper Airways by Repressing Virus-Induced Cell Cycle Arrest and Mitochondria-Dependent Apoptosis
Source: Front Immunol. 2022 Mar 31;13:872958. doi: 10.3389/fimmu.2022.872958 (PMC9009290; doi:10.3389/fimmu.2022.872958)
Supplement: Supplementary file 1 [file DataSheet_1.pdf]

Supplementary materials

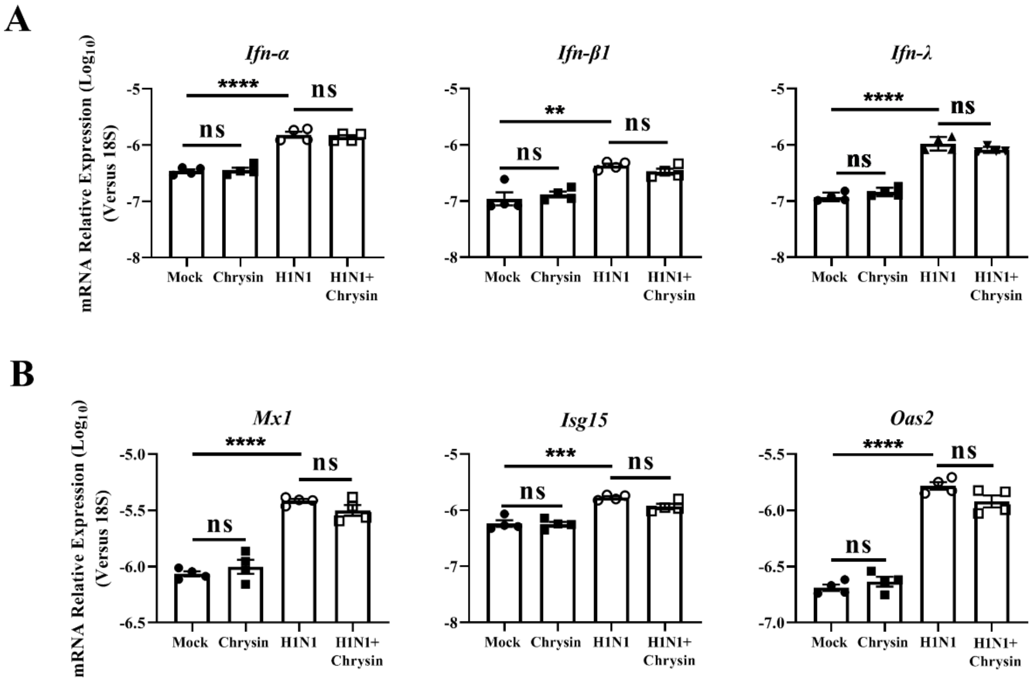

Supplementary figure 1. Chrysin did not induce the innate antiviral host response.
A549 cells were infected with or without A/PR/8/34 (MOI = 0.5) virus in the presence or absence of 8 μM chrysin for 2 hours, the medium was removed and replaced by medium containing 8 μM chrysin for 4 hours of treatment. Host mRNA expression levels of IFNs (IFNα, IFN-β1, and IFN-λ) **(A)** and ISGs (Mx1, Isg15, and Oas2) **(B)** were determined by RT-qPCR. Data are expressed as the mean ± SEM of three independent experiments with a log value. **P < 0.01, ***P < 0.001, ****P < 0.0001 by unpaired two-tailed Student's t-test. ns, no significant difference.

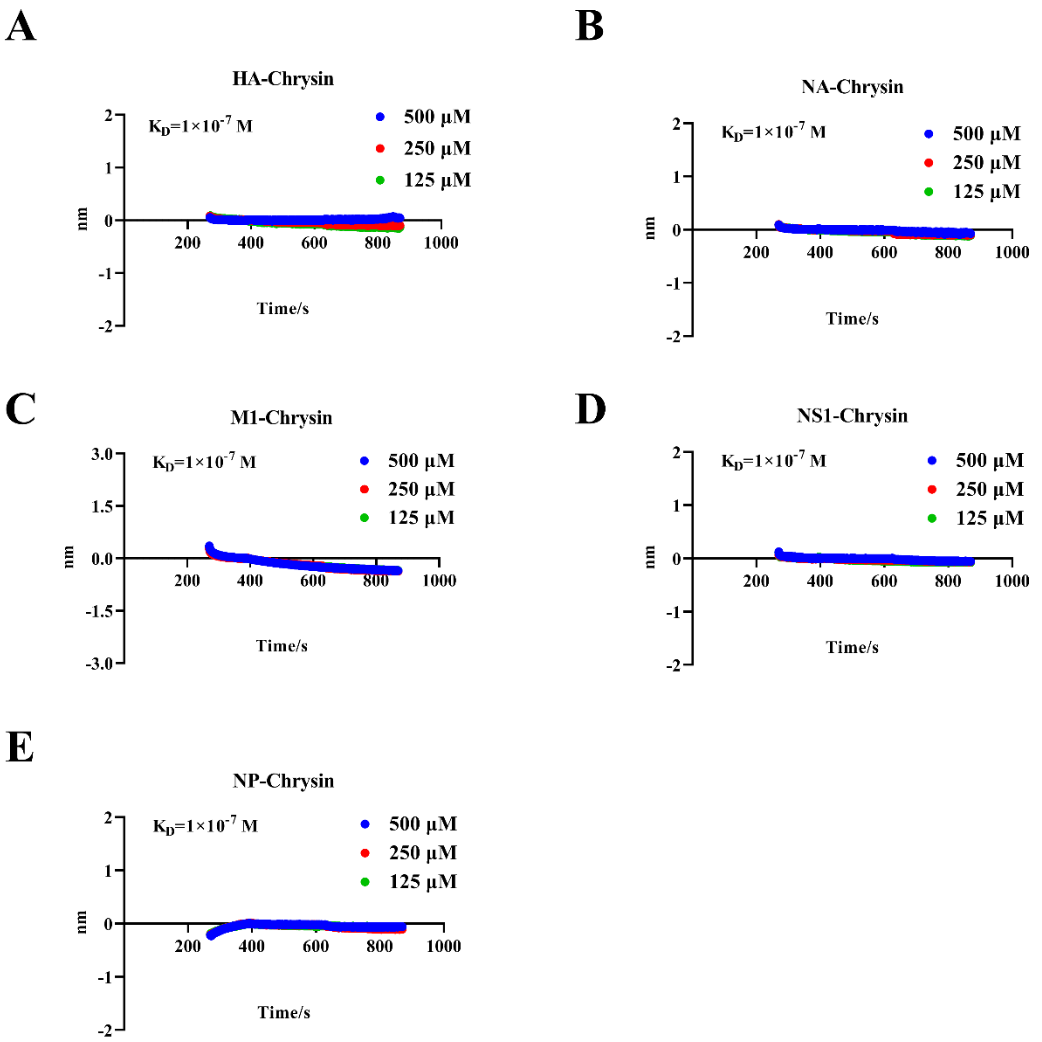

Supplementary figure 2. Characterization of chrysin interactions with H1N1 viral proteins.
H1N1 viral proteins HA **(A)**, NA **(B)**, M1 **(C)**, NS1 **(D)**, and NP **(E)** were immobilized on streptavidin sensors and reacted with the appropriate amounts of chrysin for biolayer interferometry (BLI). The apparent dissociation constants, K_D (M), were calculated and shown in the graph. Data are representative of two independent experiments.

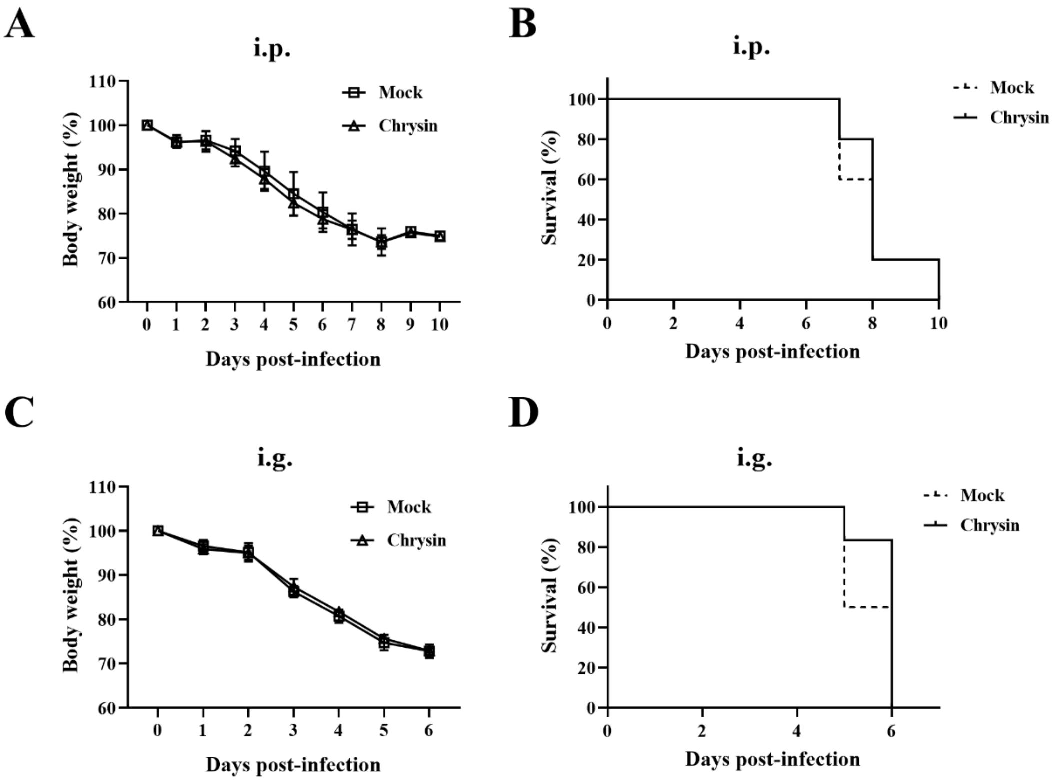

Supplementary figure 3. Intraperitoneal or intragastric administration of chrysin failed to protect mice against influenza virus infection.

(A-B) C57BL/6 mice (n = 6 per group) were administered intraperitoneally with 100 mg/kg chrysin daily after being intranasally challenged with 18 PFU A/PR/8/34 (H1N1) in a total volume of 40 µl. Weight loss (left panel) and survival (right panel) were monitored daily until the animals were sacrificed once they had reached 25% of their original body weight. **(C-D)** C57BL/6 mice (n = 6 per group) were given 100 mg/kg chrysin intragastrically daily after being intranasally infected with 18 PFU A/PR/8/34 (H1N1) in a total volume of 40 µl. Weight loss (left panel) and survival (left panel) were monitored daily until the animals were sacrificed once they had dropped 25% of their initial body weight. Data are representative of two independent experiments.
